# A Web-Based Mind-Body Intervention to Improve Resilience Among Patients With Nontraumatic Painful Upper-Extremity Conditions and Comorbid Risky Substance Use: Protocol for a Mixed Methods Study

**DOI:** 10.2196/64547

**Published:** 2024-12-09

**Authors:** Nadine Levey, Neal Chen, Joseph Ditre, Louisa Sylvia, Chaitanya Mudgal, Abhiram Bhashyam, Rohit Garg, David Ring, Ana-Maria Vranceanu, Jafar Bakhshaie

**Affiliations:** 1 Center for Health Outcomes and Interdisciplinary Research Department of Psychiatry Massachusetts General Hospital Boston, MA United States; 2 Hand and Arm Center Department of Orthopedic Surgery Massachusetts General Hospital Boston, MA United States; 3 Harvard Medical School Boston, MA United States; 4 Center for Health Behavior Research & Innovation Department of Psychology Syracuse University Syracuse, NY United States; 5 Dauten Family Center for Bipolar Treatment Innovation Department of Psychiatry Massachusetts General Hospital Boston, MA United States; 6 Dell Medical School The University of Texas at Austin Austin, TX United States

**Keywords:** chronic pain, upper-extremity conditions, psychiatry, mindfulness, mind-body, substance use, web-based intervention development

## Abstract

**Background:**

Nontraumatic painful upper-extremity conditions (NPUCs) are largely age-related degenerations that affect the majority of adults. Most patients with NPUCs do not seek medical care and adjust on their own. Among those who do seek care, approximately 20% report risky substance use, defined as a consumption pattern that increases the risk of harm to physical or psychosocial health. In the context of NPUC, risky substance use is associated with more intense pain, emotional distress, disability, and opioid or other substance misuse (ie, cross-tolerance). Consequently, risky substance use is a significant modifiable risk factor for the progression and maintenance of chronic pain-related disability and comorbid psychopathology among patients with NPUCs.

**Objective:**

This study aims to develop, adapt, and test the feasibility of the Web-Based Toolkit for Resilient Life Beyond Pain and Substance Use (Web-TIRELESS), a novel, asynchronous, and web-based mind-body intervention aimed at modifying maladaptive pain-coping behaviors in patients with NPUC and comorbid risky substance use. This study illustrates the proposed study design, methodology, and intervention content.

**Methods:**

In aim 1, we will conduct live video qualitative interviews (n=20) with care-seeking adult patients with NPUC and comorbid risky substance use to inform the development and refinement of Web-TIRELESS and study procedures. In aim 2, we propose an open pilot study (n=12) of Web-TIRELESS with exit interviews and pre- and postintervention assessments to evaluate the feasibility, credibility, and acceptability of Web-TIRELESS and refine study procedures. Aim 3 consists of a pilot feasibility randomized controlled trial of Web-TIRELESS versus minimally enhanced usual care (n=50), both of which follow a web-based modality, to demonstrate the feasibility of recruitment procedures and data collection, as well as the feasibility, credibility, and acceptability of Web-TIRELESS and the control condition (adherence, retention, fidelity, and satisfaction), following prespecified benchmarks.

**Results:**

Patient interviews (aim 1) concluded in May 2024 and qualitative analysis is expected to be completed in September 2024. Completion of aim 2 (data collection and analysis) is expected by June 2025. The completion of aim 3 and other study-related operations is anticipated by June 2027.

**Conclusions:**

We will develop and test Web-TIRELESS, the first asynchronous mind-body intervention specifically adapted to enhance resilience in response to chronic pain among individuals with NPUCs and comorbid risky substance use. Results from this 3-aim study (feasibility, acceptability, and satisfaction of Web-TIRELESS) will be leveraged to inform a future efficacy randomized controlled trial of Web-TIRELESS versus the minimally enhanced usual care.

**Trial Registration:**

ClinicalTrials.gov NCT06366633; https://clinicaltrials.gov/study/NCT06366633

**International Registered Report Identifier (IRRID):**

DERR1-10.2196/64547

## Introduction

### Background

Nontraumatic painful upper-extremity conditions (NPUCs) are highly prevalent in the adult population and are typically age-related degenerations that become chronically painful [1-3]. The most common NPUC diagnoses include trigger joints; tendinopathy; and arthritis of the shoulder, elbow, and hand [4]. Most patients with NPUCs do not seek treatment and instead find ways to independently adapt [5,6]. Of those who do seek care from a medical provider, many choose not to pursue treatment upon reassurance from their provider and learning that most interventions are palliative (eg, joint injections and pain medications) rather than disease modifying [4,7]. For patients who commit to medical treatment, outcomes can vary widely, leading to inconsistent results, and the emotional and financial costs involved are significant [4,7,8]. Therefore, it is essential to develop evidence-based interventions that address the unique needs of patients who seek care for their NPUCs.

**Figure 1:**
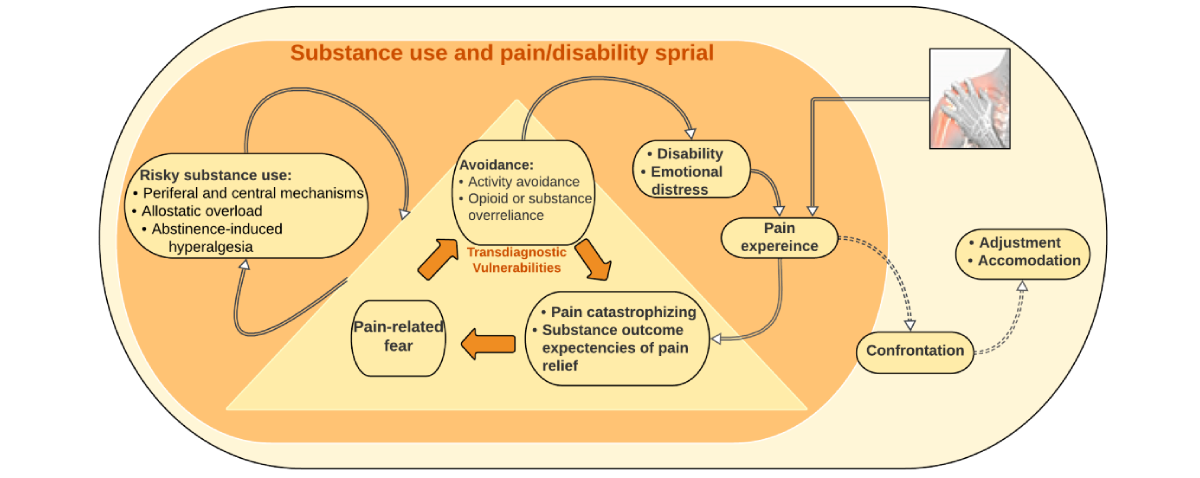
Reciprocal model of pain and substance use with adaptations from the fear-avoidance model of pain.

Risky substance use, defined as patterns of use that increase one’s risk of physical or psychological harm, is a maladaptive behavioral pain response exhibited in up to 20% of care-seeking patients with NPUC [9]. Comorbid risky substance use is associated with worse treatment outcomes [9-11], greater pain severity, disability, psychological distress, and the development and progression of chronic pain [10,12,13]. The established reciprocal model of pain and substance use can explain the development and maintenance of pain and disability in patients with NPUC and comorbid risky substance use patterns [14]. This model posits that risky substance use affects pain regulation via both peripheral (ie, tissue damage) and central (ie, aberrant pain processing) mechanisms, escalating the allostatic load on pain neurocircuitry and abstinence-induced hyperalgesia (Figure 1). This process can trigger maladaptive cognitive and behavioral responses such as pain catastrophizing, pain-related fear, and activity avoidance, which in turn can amplify the reported intensity of pain, increase disability, and exacerbate emotional distress (ie, fear-avoidance-driven cycle of pain exacerbation) [15]. According to the reciprocal model of pain and substance use, NPUC and risky substance use mutually reinforce each other via their shared transdiagnostic mechanisms of pain catastrophizing, pain-related fear, and avoidance [14,16-19]. This mechanism creates a vicious cycle that intensifies chronic pain, increases substance use, exacerbates disability, and heightens emotional distress (Figure 1) [14,20]. Therefore, it is crucial to target the underlying mechanisms driving the reciprocal relationship between pain and substance use in order to enhance treatment outcomes for this patient population.

Despite compelling evidence highlighting the detrimental impact of risky substance use on patient outcomes and health care costs, there are currently no evidence-based interventions tailored to the specific treatment needs of care-seeking patients with NPUC and comorbid risky substance use. Within the context of upper-extremity orthopedic practices, providers frequently fail to address the threat to treatment outcomes posed by patterns of risky substance use. Moreover, patients with NPUC may also minimize or fail to disclose their substance use, either to obtain prescription pain medication or due to the perceived stigma surrounding substance use [21]. These deficiencies pose a substantial public health concern, considering the strong correlation between risky substance use and heightened mortality, multimorbidity, and associated health care expenses [22,23]. It is therefore critical to develop feasible, acceptable, and effective interventions that address maladaptive pain-related regulatory behaviors and processes in patients seeking care for NPUC who have comorbid risky substance use.

Mind-body interventions show promise in effectively addressing risky substance use, chronic pain, and associated disability. They target the transdiagnostic mechanisms contributing to both conditions [5,24-27], demonstrating effectiveness in reducing pain disability and intensity [25,26], substance use urges [27], and related distress [24-26]. These interventions are increasingly appealing to orthopedic care-seeking patients as they bypass the discomfort related to stigma when pursuing treatments for substance use and mental health issues [28]. Notably, asynchronous web-based platforms are particularly well-suited for delivering mind-body treatments to patients with comorbid nonphysical symptom chronicities (NPUCs) and risky substance use [29]. These programs hold promise because they (1) address concerns related to stigma by allowing for anonymity [28]; (2) are low cost [30,31]; (3) offer accessible long-term symptom management for NPUC [32]; (4) circumvent barriers to quality care such as limited availability of skilled providers, transportation, and commitment to a particular day or time [32]; and (5) may increase self-efficacy by supporting patients’ ownership over the management of their health condition [33].

### Objectives

**Figure 2:**
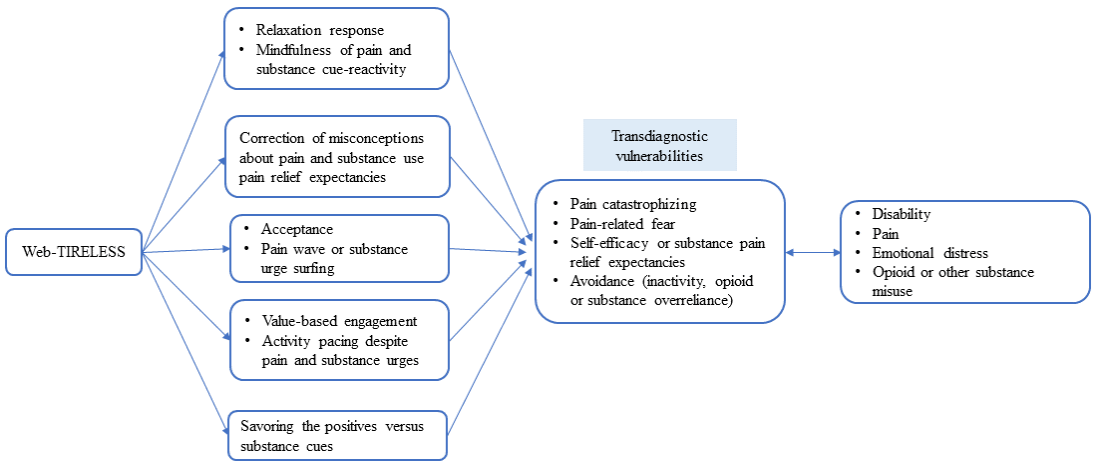
Conceptual model of Web-TIRELESS. Web-TIRELESS: Web-Based Toolkit for Resilient Life Beyond Pain and Substance Use.

**Figure 3:**
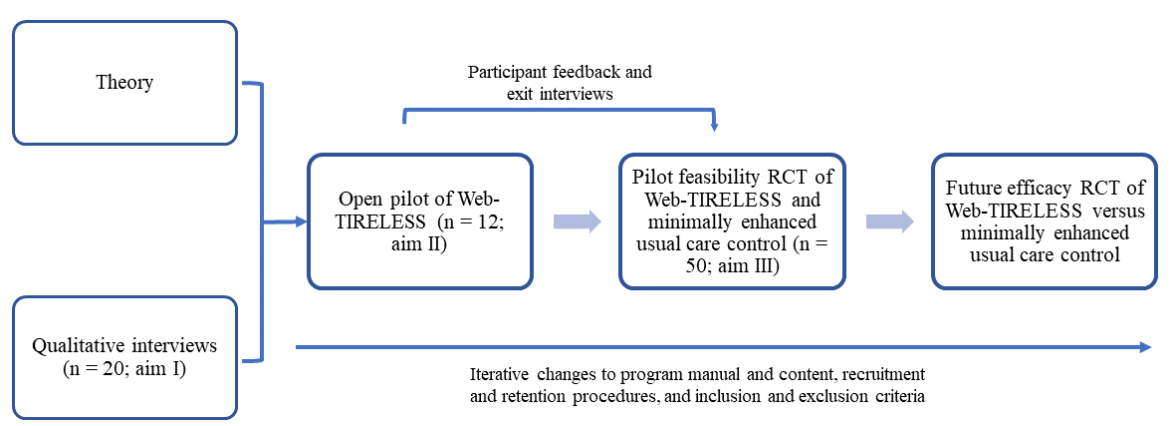
Study schema of Web-TIRELESS. RCT: randomized controlled trial; WEB-TIRELESS: Web-Based Toolkit for Resilient Life Beyond Pain and Substance Use.

This study outlines the protocol for developing and testing the feasibility of the Web-Based Toolkit for Resilient Life Beyond Pain and Substance Use (Web-TIRELESS), a web-based mind-body intervention designed for patients with NPUC and comorbid risky substance use (Figure 2). We aim to adapt the Toolkit for Optimal Recovery after Orthopedic Injury (TOR)—our team’s previously developed brief, live-video, mind-body preventive treatment for chronic pain among at-risk individuals with acute orthopedic injuries [34]—for the unique needs of our target population and evaluate its feasibility, acceptability, and credibility. We propose a mixed methods approach to iteratively refine Web-TIRELESS across three study aims (Figure 3).

- Qualitative live video interviews: Conduct up to 20 qualitative live video interviews with adult patients with comorbid risky substance use seeking orthopedic care for NPUC to identify this population’s treatment needs and preferences. These insights will inform the development of Web-TIRELESS and study procedures.
- Open pilot study: Implement an open pilot study with 12 participants (10 completers), including exit interviews and pre- and postintervention assessments, to evaluate the feasibility, credibility, and acceptability of Web-TIRELESS and study procedures.
- Pilot feasibility randomized controlled trial (RCT): Conduct a pilot feasibility RCT comparing Web-TIRELESS to web-based minimally enhanced usual care (Web-MEUC) with 50 participants (40 completers). This trial aims to demonstrate the feasibility of recruitment and data collection, as well as the feasibility, credibility, and acceptability of both the Web-TIRELESS and control conditions (adherence, retention, fidelity, and satisfaction). We hypothesize that the finalized Web-TIRELESS will be feasible; acceptable from a patient perspective; and have the potential to effectively and efficiently decrease disability, pain, risky substance use, and related distress among care-seeking patients with NPUC and comorbid risky substance use. This paper describes the study protocol and methodology.

## Methods

### Study Design

The design and methodology of this study are in alignment with the Science of Behavioral Change framework [35] and the National Center for Complementary and Integrative Health (NCCIH) guidelines for intervention development [36]. Therefore, our primary outcomes will be the feasibility, credibility, and acceptability of Web-TIRELESS, rather than efficacy. The secondary outcomes are pain intensity, disability, pain catastrophizing, and avoidance. See Multimedia Appendix 1 for peer review of this research proposal by the NCCIH.

### Ethical Considerations

The Massachusetts General Brigham (MGB) institutional review board (IRB) approved all the study procedures for aims 1 and 2 (2021P001630; 2024P000713).

### Inclusion and Exclusion Criteria

Study inclusion criteria for all study phases are as follows: (1) care-seeking patient of the Massachusetts General Hospital Orthopedic Hand and Arm Center; (2) aged 18 years and older; (3) diagnosed with NPUC (eg, arthritis or trigger finger); (4) pain score>4 on the Numerical Rating Scale [37]; (5) risky substance use (score of >10 and <27 for alcohol, or >3 and <27 for cannabis, cocaine, amphetamine-type stimulants, inhalants, sedatives, hallucinogens, opioids, tobacco, or E-cigarettes on the Alcohol, Smoking and Substance Involvement Screening Test [ASSIST]) [38]; (6) owns a device with internet access; (7) English fluency; and (8) able and willing to participate.

Exclusion criteria for all study phases are as follows: (1) participation in mind-body or specialized substance abuse treatment in the past 3 months; (2) practice of mindfulness more than 45 min/wk in the past 3 months; (3) changes to psychotropic medication (eg, antidepressants) within the last 3 months; (4) serious untreated mental illness (eg, schizophrenia); (5) current suicidal ideations; (6) pregnancy; (7) secondary gains (eg, pending disability claim); (8) moderate to severe cognitive impairment (≥4 on the Short Portable Mental Status Questionnaire) [39]; and (9) lifetime history of surgery for the presented NPUC (aim 1) or history of surgery for the presented NPUC within the last 6 months (aims 2 and 3).

### Recruitment and Sampling

All patients will be recruited and enrolled out of the Orthopedic Hand and Arm Service at Massachusetts General Hospital’s main campus. Study staff will review the medical records of patients with upcoming appointments, identify prospective participants, and inform clinic staff of their potential eligibility. On the day of their medical appointment, study staff will approach patients in person during their clinic visit to assess study eligibility and interest. Only patients confirmed by clinic staff or medical charts to have a nontraumatic condition will be considered for enrollment. To avoid interfering with clinic flow, informed consent forms (aims 2 and 3 only) will be sent and signed electronically via REDCap (Research Electronic Data Capture; Vanderbilt University), a secure web-based survey platform hosted by MGB.

### Procedures

#### Aim 1 (Development Phase)—Qualitative Interviews

The MGB-IRB approved all study procedures and determined that this phase of the study was exempt from written informed consent. First, we will develop a semistructured qualitative interview script that assesses the target population’s specific treatment experiences and needs, as well as their perceptions of Web-TIRELESS. Additionally, we will develop a Web-TIRELESS prototype to be showcased to participants for feedback. Up to 20 interviews will be conducted with eligible patients via live video. All interviews will be guided by the semistructured script and facilitated by a trained member of the study staff. Specifically, the interview topics include (1) perception of effects of the NPUC and risky substance use on function; (2) pain management and substance use cessation skills training needs; (3) difficult situations or challenging experiences; (4) perception of the Web-TIRELESS prototype; (5) barriers and facilitators to participation and adherence, including comfort and confidence working with web-based platforms, physical barriers such as difficulty typing or using mouse, and strategies to address this (eg, education, voice activation); and (6) ideal recruitment and retention strategies. Participants will be provided with information on core program content, skills, and format for their perceptions and feedback.

Interested eligible patients will provide informed verbal consent to participate and can receive assistance with operating the videoconferencing software (ie, Zoom [Zoom Video Communications]) as necessary. All qualitative interviews will be audio recorded with the patient’s permission, transcribed, and analyzed using Dedoose version 9.0.90 (SocioCultural Research Consultants). Data from the interviews will be used to further develop and refine Web-TIRELESS.

#### Aim 2 (Development Phase)— Open Pilot Study

We will conduct an open pilot of Web-TIRELESS followed by exit interviews with up to 12 patients. Participants will complete survey assessments at baseline and postintervention and encouraged to practice and log their use of program skills regularly. This information will provide evidence of the degree to which participants are engaged with the program platform and content. Each of the 4 Web-TIRELESS sessions will be between 30 and 45 minutes long. The exit interviews will take approximately 30 minutes and will follow similar procedures to aim 1 (eg, audio recorded and transcribed). A semistructured interview script will be developed to assess participants’ perceptions of the program skills, methodology, feasibility, acceptability, and satisfaction. Results from the open pilot and exit interviews will be used to further refine the study procedures and Web-TIRELESS program. The *Data Analysis* section provides a detailed description of the analysis plan.

#### Aim 3 (Intervention Phase)—Feasibility RCT

Following written informed consent and the baseline assessment, up to 50 participants will be randomly assigned via permuted blocks using a sequence developed by a statistician to either the active (Web-TIRELESS) or control (Web-MEUC) program on a 1:1 ratio. Both interventions will be web based, and participants will be blinded to the study conditions. All patients will also complete survey assessments at baseline, posttest (ie, after completing the 4-week active or control intervention), and 6-month follow-up via the secure REDCap system.

### Program Structure and Modification

#### Web-TIRELESS (Intervention)

The original TOR follows a 1-on-1, live-video modality and is composed of 4 manualized 45-minute weekly sessions with a clinician. TOR aims to teach mind-body skills that elicit the relaxation response (eg, body scan, deep breathing), mindfulness, cognitive-behavioral strategies (eg, reframing), activity pacing, and acceptance and commitment skills (eg, acceptance) [34]. In the process of adapting TOR, we will remove the irrelevant skills, revise the language to focus on upper extremity conditions, and add new modules relevant to resilience in the face of chronic pain and substance use (see Table 1 for the proposed Web-TIRELESS content). The Web-TIRELESS content may be subject to minor changes following the integration of participant feedback. The modality of Web-TIRLESS will differ from TOR in that all sessions will be on-demand 30 to 45-minute web-based videos. Each Web-TIRELESS session will consist of short (1-5 minutes) educational, exercise, and home practice goal-setting videos. Participants will be instructed to complete 1 session per week on a day and time of their choosing. While participants are given guidance on how to leverage Web-TIRELESS as a resource, session frequency and overall engagement with the program will be determined by each individual. The end of each session includes a “prescription for resilience,” which provides specific home practice assignments for the following week (eg, “Practice deep breathing at least once this week”). Following the completion of each session, participants must pass a knowledge check (ie, a 5-minute web-based quiz) before proceeding to the next session. In between sessions, participants are encouraged via text message reminders to practice and log their use of the Web-TIRELESS program skills independently. Home practice materials will include audio and video clips from the most recent session that guide the independent practice of program skills. The overall duration of the program will vary per participant but is expected to take 3-5 weeks to complete.

**Table 1:** Proposed adaptations for the Web-TIRELESS^a^ intervention.

Session	Original toolkit (TOR^b^)	Adapted content for NPUC^c^ and web-based delivery (Web-TIRELESS)
—^d^	—	Introductory information session on the Web-TIRELESS program (eg, how to use it, where to get support).
1	Treatment rationale Physical, emotional, and cognitive factors that impact recovery after injury Debunk myths about pain Set goals for recovery Relaxation strategies Set homework	Education Treatment rationale for targeting pain or disability and promoting resilience or adaptation Education on the spiral of pain, disability, and substance use and its risk factors Debunk pain misconceptions and substance use outcome expectancies of pain relief Introduce relaxation response strategies for managing pain and substance use urges Skills Goal setting for managing pain and substance use Scripted breathing and body scan practices Establish goals for home practice Exercise 2-minute diaphragmatic breathing 5-minute web-based quiz
2	Review homework Discuss biopsychosocial model and mind-body links for pain Develop the patient’s unique recovery path. Mindfulness skills for habituating to pain Set homework	Review (following 5 minutes of scripted breathing practice) Home practice; tips for problem-solving barriers to practice. Prompts for reflection Education “pain spiral” and “coping path” Provide education on biopsychosocial model and mind-body links for NPUC or substance use. Psychoeducation on urge lifecycle and dynamics and ways to fight against impulses. Skills Develop personalized pain or coping path and substance use cessation. Pain or substance urge wave surfing exercise focusing on the transitory nature of episodes. Mindfulness skills for habituating to NPUC and substance use urge time-course (mindful STOP^e^; mindful breathing) Home practice goal setting Exercise 5-Minute “Substance Use Urge Surfing” video to facilitate substance use impulse control. 5-minute web-based quiz
3	Review homework Review personal recovery path Mindfulness of pain Challenge unhelpful thoughts about pain to speed up recovery Learn behaviors to speed up recovery (activity pacing) Set homework	Review (following 5 minutes of scripted breathing practice) Home practice; tips for problem-solving barriers to practice. Prompts for reflection Education Mindfulness of pain and substance use urges. Psychoeducation on negative automatic thoughts (“unhelpful thoughts about pain, and substance-related expectancies: pain catastrophizing, fear and avoidance”) and cognitive reframing Physical activity pacing and setting activity goals Psychoeducation on positive affective responses to natural reward versus substance cues (“Savoring the positives”). Skills Observer exercise for pain and substance use urges Challenge unhelpful thoughts (ie, cognitive reframing) Savoring the positive technique to increase resiliency Home practice goal setting Exercise 5-minute “Observer Exercise” video to facilitate self-identity beyond pain and substance use. 5-minute web-based quiz
4	Review homework Acceptance strategies Review all toolkit skills Review challenges with toolkit Plan for continued coping	Review (following 5 minutes of scripted breathing practice) Home practice; tips for problem-solving barriers to practice Prompts for reflection Education Acceptance strategies for pain, substance urge, and related distress. Reframing versus acceptance for pain and substance urges. Tips for staying on the coping path and implementing skills in the future (eg, pain communication, social support seeking, sleep hygiene, recognizing high-risk substance use situations like pain flare-ups). Referral information Skills Acceptance-based exercises (eg, diffusion, self as context, and self-compassion strategies). Review all program skills Exercise 2-minute “Ladder Metaphor” video to help patients plan for possible barriers to resilience in the future (ie, relapse prevention). 5-minute web-based quiz

^a^WEB-TIRELESS: Web-Based Toolkit for Resilient Life Beyond Pain and Substance Use.

^b^TOR: Toolkit for Optimal Recovery after Orthopedic Injury.

^c^NPUC: nontraumatic painful upper-extremity condition.

^d^Not applicable.

^e^STOP: Stop, Take a breath, Observe with curiosity, Proceed mindfully.

#### Web-MEUC (Control)

The Web-MEUC will be an educational digital web-based pamphlet (accessible for the duration of follow-up) containing brief information related to the intervention topics. This includes the trajectory of pain and adaptation in NPUC, the role of relaxation strategies to manage pain, the impact of maladaptive coping behaviors such as substance use, and the importance of cessation and returning to engagement in activities of daily living. Web-MEUC will not include any skills (ie, active ingredients).

### Measures

#### Sociodemographic Characteristics (Baseline Only)

To better understand our sample, we will collect the following sociodemographic information at baseline: age; sex; gender identity; race; ethnicity; education level; marital status; and employment status.

#### Clinical Variables (Baseline Only)

We will collect data on NPUC, date of diagnosis, symptom trajectory, and treatment history.

#### Involvement of Substance Use (Eligibility Screening Only)

To assess the severity of risky substance use, we will use the World Health Organization’s ASSIST [38]. Each substance type (ie, alcohol, tobacco, cannabis, stimulants, inhalants, sedatives, hallucinogens, and opioids) is scored separately on the ASSIST, with scores on each ranging from 0 to 40. Higher scores indicate more problematic substance use for the respective substance type.

### Primary Outcomes

In accordance with the Science of Behavioral Change framework [35] and NCCIH guidelines for intervention development [36], primary outcomes for this 3-aim study include a priori feasibility, credibility, fidelity, and credibility benchmarks (Table 2).

- Feasibility of recruitment: The proportion of eligible patients that agree to participate.
- Treatment credibility (baseline only): The proportion of participants that score above the midline on the Credibility and Expectancy Questionnaire [40]. The Credibility and Expectancy Questionnaire will be administered to assess the degree to which one believes the intervention will effectively manage one’s upper extremity pain condition, substance use, and related worries. Some items are scored on an 11-point 0%-100% scale while others are scored on a 1-9 Linkert scale. Higher scores represent higher credibility and expectancy.
- Feasibility of assessments: The proportion of participants that complete survey assessment with no measure fully missing.
- Acceptability of treatment: The proportion of participants that complete 3 of 4 program sessions.
- Feasibility of homework adherence: The proportion of participants that practice and report using 1 or more skills on 3 d/wk.
- Treatment satisfaction (postintervention time point only): The proportion of participants that score above the midline on the Client Satisfaction Questionnaire [41]. The Client Satisfaction Questionnaire is a 3-item questionnaire that measures on a scale of 1 to 4 the degree to which the participant is satisfied with the program and its ability to meet their needs. Higher scores represent greater satisfaction.
- Acceptability of web-based delivery (postintervention time point only): The proportion of participants that score above the midline on the User Experience Scale [42]. The User Experience Scale assesses how well patients like the web-based program content and delivery. Total scores range from 22 to 110 with higher scores indicating greater satisfaction with the platform.

**Table 2:** A priori feasibility benchmarks.

Outcome	Acceptable	Excellent
Credibility and expectancy	70% and more of participants with score over the midpoint on the Credibility and Expectancy Scale	75% and more of participants with score over the midpoint on the Credibility and Expectancy Scale
Acceptability of treatment (satisfaction)	70% and more of participants with scores over the midline on the Client Satisfaction Scale	75% or more of participants with scores over the midline on the Client Satisfaction Scale
Acceptability of web-based delivery (satisfaction)	70% and more of participants with scores over the midline on the User Experience Scale	75% and more of participants with scores over the midline on the User Experience Scale
Feasibility of recruitment	70% and more eligible patients approached agree to participate	80% and more of eligible patients approached agree to participate
Acceptability of treatment (attendance)	70% and more of participants attend 3 out of 4 sessions	80% and more of participants attend 3 out of 4 sessions
Adherence to homework	70% and more of participants practice at least 1 skill on 3 days/week	80% and more of participants practice at least 1 skill on 3 days/week
Feasibility of assessments	70% and more of participants complete all assessments (with no measures missing)	90% and more of participants complete all assessments (with no measures missing)
Adverse events	Minimal	None

### Secondary Outcomes

Participants in the open pilot and feasibility RCT will complete the surveys described later at baseline, postintervention (ie, after fully completing active or control intervention), and a 6-month follow-up (feasibility RCT only) unless otherwise stated.

#### Pain Severity

The Graded Chronic Pain Scale [43] will be used to assess pain severity and pain-related disability. Total scores range from 0 to 10 with higher scores indicating worse pain severity.

#### Pain Intensity

The Numerical Rating Scale [37] will assess the intensity of pain at rest and with activity on a 10-point Likert scale (eg, from 0=no pain to 10=worst possible pain).

#### Disability

The 30-item Disabilities of the Arm, Shoulder, and Hand [44] will quantify the severity of disability as it relates to NPUC. Higher scores indicate greater disability.

#### Pain Catastrophizing

The Pain Catastrophizing Scale [45] will be administered to assess participants' proclivity for magnification, helplessness, and rumination of pain. Scores range from 0 to 52 with higher scores indicating greater pain catastrophizing.

#### Pain Anxiety

We will use the Pain Anxiety Symptoms Scale–Short Form [46] to assess fear and anxiety related to pain. Scores range from 0 to 100 with higher scores indicating greater pain-related fear and anxiety.

#### Hypervigilance

The Pain Vigilance and Awareness Questionnaire [47] will measure participants’ preoccupation with or attention to pain. Scores range from 16 to 96 with higher scores indicating greater pain vigilance.

#### Fear Avoidance

We will assess fear avoidance using the Brief Experiential Avoidance Questionnaire [48] which quantifies tendencies to avoid unpleasant internal experiences. Scores range from 15 to 90 with higher scores indicating greater avoidance behaviors.

#### Opioid Misuse

Participants will complete the Current Opioid Misuse Measure [49], which assesses the risk for aberrant medication-related behavior in persons with chronic pain. Scores range from 0 to 68 with higher scores indicating greater risk for opioid misuse.

#### Substance Use

The Timeline Follow Back [50] will be self-administered by patients following the completion of each Web-TIRELESS session and leveraged as a motivational interviewing technique. The Timeline Follow assesses daily estimates of alcohol, cannabis, cigarette, and other substance use during the past week (0 to 7 days prior). Higher scores indicate more severe substance use.

#### Anxiety

To assess symptoms of anxiety, we will use the 8-item Patient-Reported Outcomes Measurement Information System Emotional Distress–Anxiety 8b version 1.0 [51]. Scores of this measure range from 8 to 40 with higher scores indicating greater anxiety symptom severity.

#### Depression

The Patient-Reported Outcomes Measurement Information System Emotional Distress–Depression 8b version 1.0 [51] is an 8-item measure that will be used to assess symptoms of depression. Scores range from 8 to 40 with higher scores indicating greater depression symptom severity.

### Self-Report Measures

Patients will be required to fill out a series of questionnaires online through the secure REDCap system. The completion of these questionnaires is expected to take approximately 30 minutes. To enhance data completeness, patients will be prompted to address any missed items at the end of the session. Additionally, a blinded research assistant, who is not involved in the recruitment process, will review each completed questionnaire to confirm that responses are not randomly made or uniformly affirmative.

### Data Analysis

#### Aim 1: Qualitative Interviews

Analyses will be guided by the framework method [52,53] and follow a deductive-inductive approach with predetermined themes [54] while allowing for inductive flexibility where themes or codes come from data [52,55]. Prior to initiating the qualitative coding, we will develop a preliminary codebook with predetermined themes [54], which will be subject to changes as distinct patterns in the data emerge throughout coding [52,55]. Qualitative data (ie, deidentified interview transcripts) will be analyzed using Dedoose version 9.0.90 (SocioCultural Research Consultants). Qualitative coding will be completed by the principal investigator and reliability coding will be done by a trained research assistant. To ensure scientific rigor, we will assess the reliability (κ) of the application of codes to qualitative responses with the ultimate goal of establishing adequate interrater reliability (κ>.80). Discrepancies in the application of codes will be resolved through discussion until agreement is sufficiently reached.

#### Aim 2: Open Pilot Study

We will use a mixed methods approach [56] to analyze the quantitative survey data and qualitative exit interview data. For quantitative analysis, we will focus on descriptive statistics with estimated variance for each measure, and within-group pre-post paired 2-tailed *t* tests (with Cohen *d* effect sizes). This will allow us to assess the preliminary efficacy of Web-TIRELESS. We will also execute exploratory correlations to test the processes (eg, within-group changes in risky substance use and pain or disability) [57-59]. Frequency and proportions will also be used to assess the feasibility of recruitment, retention, and treatment credibility or satisfaction (Table 2). Qualitative data derived from exit interviews will be analyzed using the procedures outlined in aim 1. Finally, we will use the embedded approach for mixed methods [56] to integrate the qualitative and quantitative data and interpret the results to refine the Web-TIRELESS before the feasibility RCT.

#### Aim 3: Feasibility RCT

We will use the same analysis techniques to assess feasibility, acceptability, credibility, and satisfaction as in aim 2. Drop-outs will be counted as not meeting applicable feasibility criteria. Benchmarks that need to be met before an efficacy trial (Table 2) will be reported separately for each intervention [60]. If these criteria are not met, we will make the necessary revisions to ensure that they are fulfilled.

#### Treatment Fidelity

For aims 1 and 2, fidelity to the web-based program material will be assessed using a user experience software (eg, Google Analytics [Google]) that monitors engagement with the web-based treatments (access to and time spent on each web page, log-in and use of material, and completion of web-based quizzes).

#### Power Analysis

This trial is primarily focused on feasibility and not efficacy, meaning that power analysis of group differences or significant pre-post changes in outcome measures is inappropriate. Furthermore, power analysis is not appropriate for qualitative analysis. However, saturation of themes is expected for the qualitative interviews with up to 20 patients [61]. With a sample size of 50 in aim 3 and assuming conservatively that the 7 feasibility criteria evaluated are independent, the study will have more than 80% power to confirm all feasibility criteria if the expected rate of each criterion is at least 83%.

## Results

This study is funded by the National Institutes of Health (1K23AT012364-01). Aim 1 was approved by the MGB-IRB (P# 2021P001630) in January 2023, qualitative interviews concluded in May 2024, and completion of aim-1 data analysis is expected in December 2024. MGB-IRB protocol approval was garnered for aim 2 (P# 2024P000713) in May 2024 and recruitment started in October 2024. Completion of aim-2 data analysis is projected for June 2025. Aim-3 IRB approval and initiation of participant recruitment is anticipated by July 2025. Data collection and analysis (aim 3) are expected to conclude in January 2027. Overall completion of aim 3 and other study-related operations is anticipated by June 2027.

## Discussion

### Significance

Risky substance use is common among care-seeking patients with NPUC, with prevalence rates of up to 1 in 5. It serves as a significant modifiable risk factor for the progression and maintenance of chronic pain, opioid and other substance use disorders, disability, and distress [9,10,12-14]. Identifying patients with NPUC and comorbid risky substance use and enrolling them in an asynchronous, web-based mind-body program may be an effective and efficient approach to reducing pain and disability while preventing prolonged and costly care for this population. In this paper, we illustrate the methodology and study procedures for developing, adapting, and establishing the feasibility of Web-TIRELESS, a novel program aimed at enhancing resilience and fostering adaptive coping strategies for chronic pain, substance use, and related distress among care-seeking patients with NPUC and comorbid risky substance use. Informed by the reciprocal model of pain and substance use [14] as well as the fear-avoidance model of pain [15,62], Web-TIRELESS proposes an integrated approach. This approach harnesses a range of mind-body skills (eg, mindfulness, deep breathing, savoring the positives), cognitive behavioral strategies (eg, reframing), acceptance and commitment skills (eg, substance use urge surfing, value-based goal setting). These components are strategically designed to target transdiagnostic factors relevant to NPUC and the comorbidity of risky substance use.

The results of this 3-aim trial will offer crucial insights to prepare for a fully powered efficacy RCT comparing Web-TIRELESS with a Web-MEUC control. This design and methodology align with the Science of Behavioral Change framework [35] and NCCIH recommendations for intervention development, which posit that iterative refinement and establishment of feasibility best ensure the scientific rigor of subsequent efficacy trials [36]. Designing and developing behavioral interventions and study procedures that preemptively identify prospective patients, cater to the specific needs of the target population, and establish the power to detect change is vital to safeguard against the common consequences of testing efficacy before feasibility. Importantly, this methodical process elucidates valuable firsthand information from the target population (eg, treatment needs, preferences, perceptions, and barriers to treatment), which may inform the development of other treatments or interventions tailored to this population. If our proposed model of care proves effective, it could be extended to other patient populations with nontraumatic painful conditions, such as those affecting the knee, hip, ankle, or spine, who present with similar treatment challenges.

### Limitations

The primary limitation of this study includes its limited scope. Given that the primary outcomes of each study aim are centered around feasibility, we cannot make claims of program efficacy until a future efficacy trial. Additionally, because the program is currently being developed for English speakers only, the generalizability of our results across diverse ethnolinguistic groups will be unknown. Finally, due to our sample consisting of only patients with NPUCs, it is unclear if the effects will be comparable across other patient populations with nontraumatic painful conditions and risky substance use.

### Conclusions

In summary, this study aims to develop, adapt, and establish the feasibility of Web-TIRELESS, the first asynchronous mind-body intervention specifically tailored to aid in coping with chronic upper-extremity pain among individuals with NPUCs and comorbid risky substance use. By iteratively refining and adapting the intervention based on feedback from qualitative patient interviews, we aim to increase the likelihood of establishing program efficacy in future trials. The results from this study will inform a future efficacy RCT of Web-TIRELESS versus a Web-MEUC control group. Future studies should also explore the extent to which Web-TIRELESS can be applied to comparable patient groups experiencing both risky substance use and nontraumatic painful conditions.

## Appendix

Multimedia Appendix 1 Peer review report from the National Center for Complementary and Integrative Health Special Emphasis Panel (ZAT1 JM (13)) - NCCIH Training and Education Review Panel (National Institutes of Health, USA).

## Abbreviations

ASSIST: Alcohol, Smoking and Substance Involvement Screening Test
IRB: institutional review board
MGB: Massachusetts General Brigham
NCCIH: National Center for Complementary and Integrative Health
NPUC: nontraumatic painful upper-extremity condition
RCT: randomized controlled trial
REDCap: Research Electronic Data Capture
Web-MEUC: web-based minimally enhanced usual care
Web-TIRELESS: Web-Based Toolkit for Resilient Life Beyond Pain and Substance Use

## Data Availability

Upon trial completion, the datasets generated during or analyzed during this study are available from the corresponding author on reasonable request.
