# Supplementary material for: A Web-Based Mind-Body Intervention to Improve Resilience Among Patients With Nontraumatic Painful Upper-Extremity Conditions and Comorbid Risky Substance Use: Protocol for a Mixed Methods Study
Source: JMIR Res Protoc. 2024 Dec 9;13:e64547. doi: 10.2196/64547 (PMC11667126; doi:10.2196/64547)
Supplement: Multimedia Appendix 1 [file resprot_v13i1e64547_app1.pdf]

1K23AT012364-01 Bakhshaie, Jafar

RESUME AND SUMMARY OF DISCUSSION: This K23 application titled “Web-based mind-body treatment to enhance resilience among patients with painful nontraumatic upper extremity conditions and comorbid risky substance use” is submitted in response to PA-20-206 “Mentored Patient-Oriented Research Career Development Award (Parent K23 Independent Clinical Trial Required)” by Massachusetts General Hospital (MGH) with Dr. Jafar Bakhshaie as the candidate. This application aims to develop, refine, and test the feasibility and acceptability of a brief, integrated mind-body intervention, delivered via an asynchronous web-based platform, to support patients with painful nontraumatic upper extremity conditions and comorbid risky substance use. Dr. Bakhshaie has a strong research background in substance use treatments and mind-body interventions that is relevant to the application and could support his ability to successfully carry out the proposed study. The candidate’s commitment and dedication to a research career are demonstrated through his strong record of productivity with over 100 publications, many of which are as first author, and several are from collaborations with his current mentors. In addition, his reference letters highlight the candidate’s motivation and potential for development as an independent investigator. The mentorship team also has high academic productivity and complementary and relevant expertise in mind-body interventions, pain, and substance use as well as economic analysis, which are needed to support the candidate’s career development goals and the proposed research. However, it is also noted that the mentorship team could have been strengthened by the inclusion of an expert in qualitative and mixed methods design. The environment at MGH is outstanding, with the appropriate resources like the Osher Center for Integrative Medicine and the Harvard Catalytic Center, to support not only the proposed research, but also the career development of the candidate. In general, the career development plan is well described to provide training in mind-body clinical trials in orthopedic populations, asynchronous digital interventions, economic evaluation, and qualitative and mixed methods. A combination of extensive coursework and training provided by his mentors would help accomplish development in these areas. The proposed career development plan is based on his prior research background and would potentially serve as vehicle to achieve his goal of transitioning to an independent clinical researcher career. However, given the extensive research experience of the candidate, some questions are raised regarding the extent to which the candidate needs additional training. In addition, some of the identified gap areas seem to overlap, bringing into question whether the career development plan could be accomplished faster than the proposed five-year timeline. At large, the research plan is well designed, and addresses the significant health issue of pain and risky substance use in an orthopedic population. Notably, the adaptation of an existing intervention into a brief mind-body program focusing on upper extremities pain syndromes is viewed as innovative. It is also well integrated with the career development plan, builds upon the candidate’s prior research experience, and is well aligned with his future career goals. However, several issues were also noted in the research plan as presented. For example, the rationale for targeting patients with painful nontraumatic upper extremity conditions with comorbid risky substance could have been better justified. Moreover, the target population is not well characterized, and better supported prevalence data is needed. It is also unclear how the intervention would be tailored and refined for the target population. In addition, while it is mentioned that the proposed asynchronous telehealth intervention would help with accessibility and to decrease stigmatization, its choice over other type of interventions is not well justified. Further, there is also a concern regarding the development of a telehealth version of this intervention and the accompanying health economic analysis at this time given the current stage of development. While the proposed timeline follow-back self-report measures to assess substance use seem appropriate, the addition of a more objective confirmation of substance use (e.g., urine drug testing) could be more reliable. Finally, the heterogeneity of substance use is not well considered in the assessment of the target population or in the design of the intervention. In conclusion, this K23 application is from an exceptional and highly productive candidate with an excellent mentorship team and institutional support. While there are some questions regarding the necessity of additional training, the overall prevailing view is that the career development and research plans would be of value and further this highly promising candidate’s

progression towards an independent clinical research career. Additional issues in the study design are considered addressable and did not significantly dampen the high enthusiasm for this application.

DESCRIPTION (provided by applicant): This K23 proposal details a comprehensive 5-year training program that will support the candidate's transition toward an independent clinical research career focused on the development and rigorous testing of scalable, technology-enhanced cost- effective, mind-body interventions for orthopedic and other costly musculoskeletal conditions. In this application, the candidate proposes a significant and innovative proposal that is directly tied with his proposed training and career development goals. Background: Painful nontraumatic degenerative upper extremity conditions are common and risky substance use commonly occurs in these conditions. When these co-occur, there is an increased risk for higher pain and disability, which are costly and challenging to treat. To date, there are no evidence-based interventions focused on adapting to these painful conditions. The Toolkit for Optimal Recovery (TOR) is a brief, live video mind-body program aimed at preventing chronic pain in patients with acute injuries, which is amenable for adaptations for the needs of patients with painful nontraumatic degenerative upper extremity conditions and risky substance use. Specific aims and research design: The proposed study aims to 1) identify treatment needs and preferences of adult (18+) new patients with non-traumatic painful, upper extremity conditions with risky substance use who seek care in the outpatient orthopedic departmentent, via live video focus groups (N=3 groups; up to N=20 patients) to inform the development of the asynchronous Web-based TIRELESS and study procedures; 2) conduct an open pilot to explore initial feasibility, acceptability, and credibility of Web-TIRELESS and study methods (N=l2 patients) using pre/post- tests and exit interviews; 3) conduct a feasibility RCT of Web-TIRELESS vs. Web-MEUC (N=50) to determine 1) feasibility of recruitment, screening, eligibility, enrollment, randomization, and assessment processes, and 2) feasibility and acceptability of the Web-TIRELESS and Web-MEUC; 3) feasibility of methodology to collect 6 months post-treatment data on medical care utilization for both groups. Findings will inform an efficacy trial through the RO1 mechanisms. Training and mentoring: Study aims are supported by 4 main training goals aimed at developing expertise in l) qualitative and mixed-methods research skills (Vranceanu); 2) mind-body clinical trials in orthopedics (Vranceanu, Ring, Chen, Ditre); 3) Web-based platforms and mHealth approaches (Sylvia, Vranceanu); and 4) economic evaluation methodology and analyses (Levy). The multidisciplinary team of the mentors will oversee the progress of the project and provide 1) a rich institutional environment, and 2) targeted coursework, scientific meetings, seminars and planned publications. Relevance to the NCCIH mission: This K23 proposal is in line with NCCIH's funding priorities of "research on whole person health," and "complementary and integrative management of pain". Impact: Collectively, the experience gained during this award will serve as the foundation for the applicant's independent career to start a line of research focused on the development and testing of in scalable, technology-enhanced cost-effective, mind- body interventions for orthopedic and comorbid substance use conditions.

PUBLIC HEALTH RELEVANCE: The proposed study will establish the feasibility, acceptability, and credibility of a novel, web-delivered mind-body program, "Toolklt for REsilient Life bEyond pain and SubStance use- TIRELESS," aimed at adapting to pain and disability among patients with painful nontraumatic upper extremity conditions who have comorbid substance use. This sub-group of patients with painful nontraumatic upper extremity conditions experience greater levels of pain and disability, opioid misuse, and medical care utilization, and poor outcomes after medical treatments. A brief, tailored mind-body program delivered to this high-risk population has the potential to decrease risky substance use, improve adaptation to pain and disability, and, in turn, reduce the utilization of orthopedic care services and costs.

CRITIQUE 1

Candidate: 1
Career Development Plan/Career Goals /Plan to Provide Mentoring: 2

Research Plan: 4
Mentor(s), Co-Mentor(s), Consultant(s), Collaborator(s): 2
Environment Commitment to the Candidate: 1

Overall Impact: The candidate, Dr. Bakhshaie, has a unique background and relevant experience to be successful in being an independent research scientist. The candidate was first trained as a medical doctor (MD degree) in Iran, then completed his MA degree and PhD degree in clinical psychology (with a minor in biostatistics) at the University of Houston, an internship and post-doctoral fellowship in clinical psychology at Baylor University, and a subsequent postdoctoral fellowship at Massachusetts General Hospital (MGH) where he is currently serving as a staff psychologist transitioning to an assistant professor. The candidate has published over 130 publications, with several as first author. He has clearly described his career objectives of becoming an independent research scientist in the field of pain and substance use disorders (SUD) and developing web-based interventions for patients with pain and SUD building on his past and current experiences and training. He has established an impressive and well-rounded mentoring group to provide him the support and guidance needed. Moreover, he has strong letters of recommendation and institutional support. His proposed research project is to develop a web-based asynchronous mind-body program to improve quality of life (QOL) in patients with non-traumatic upper extremity pain conditions and concomitant substance misuse. This target population tends to use healthcare services more frequently, have higher rates of disability and healthcare utilization, and report higher pain intensity. The intervention for this trial, TIRELESS, is based on the reciprocal model of pain and substance use and the fear avoidance model and adapted from a vetted intervention, the Toolkit for Optimal Recovery (TOR) after injury, developed by his mentors (Drs. Vranceanu and Ring) and for which he has experience with. The candidate proposes adapting TOR to be delivered asynchronously, not by a trained therapist, and focusing on pain and risky substance use. He proposes to conduct focus groups using qualitative methods, followed by an open pilot, and a pilot randomized controlled clinical trial (RCT) comparing the intervention to usual care control. He provides a practical and theoretical basis for the potential success of this project and the results could provide preliminary data to apply for a R01-level award and conduct a full RCT. The enthusiasm for this application was tempered due to some considerations in the research plan and career development program. For example, substance misuse is poorly defined. The Current Opioid Misuse Measure (COMM) assessment tool is proposed to assess substance misuse, but the COMM only assesses risk of aberrant drug taking behavior which may or may not assess misuse. The timeline follow-back (TLFB) measure is appropriate but self-reported, so the design is lacking more objective markers of substance misuse such as a review of the electronic medical record (EMR), urine drug testing, or the State Prescription Drug Monitoring Program (PDMP). The target population is poorly described with respect to prevalence; in other words, it is unclear if there a large enough population with non-traumatic upper extremity (UE) pain and co-morbid substance misuse to warrant further study. The career development plan is well detailed noting that the gaps in learning include qualitative and mixed methods research skills, conducting a mind-body trial in orthopedic populations, developing asynchronous web-based platforms and mobile (m)Health approaches and gaining expertise in economic evaluation methodology and analysis, some of which overlap and would not seem to require the proposed 5-year timeline to accomplish. Also, the addition of a mentor who is an expert on qualitative and mixed methods would have enhanced the mastering of these skills.

1. Candidate:
Strengths

- Dr. Bakhshaie has a very unique background and was first trained as a MD in Iran, then completed his MA and PhD degrees in clinical psychology (with a minor in biostatistics) at the University of Houston, an internship and post-doctoral fellowship in clinical psychology at Baylor University, and a subsequent post-doctoral fellowship at MGH where he is currently serving as a staff psychologist transitioning to an assistant professor.

- The candidate has published extensively in high-impact journals, many with his proposed mentors (Drs. Vranceanu, Ditre, and Ring) and has several first-author citations. A number of publications are in the area of mind-body interventions in musculoskeletal disorders and SUD which align with the proposed career path.
- He has served as a research project manager/study clinical supervisor for a National Center for Complementary and Integrative Health-funded (NCCIH) U grant award and has been a principal investigator on a small Harvard Medical School fellowship grant award. This has provided him with the experience in project management, recruitment, and data management. He also had training in biostatistics during his PhD degree training. These experiences provide a solid basis for successfully completing the proposed project and supporting him in becoming a productive independent investigator.
- He has very strong letters of support from his proposed mentors (Drs. Vranceanu, Ditre, Ring, Sylvia, Levy, and Chen) and institutional support from Dr. Fava, the Vice Chair of the MGH Executive Committee on Research.
- His experience during his training and close mentorship with Dr. Vranceanu has provided him with the core skills to successfully organize and conduct the proposed project.
- He has clearly articulated career development goals, including acquiring skills in qualitative and mixed methods, mind-body clinical trials, and developing skills in web-based intervention design.

Weaknesses

- No major weaknesses noted.

2. Career Development Plan/Career Goals & Objectives:

Strengths

- The candidate provides a strong rationale regarding the strengths of his background and deficiencies in core areas: qualitative and mixed methods research skills, conducting a mind-body trial in orthopedic populations, developing asynchronous web-based platforms and mHealth approaches, and gaining expertise in economic evaluation methodology and analysis. His previous background and proposed career development plan are consistent with his stated career goals of becoming an independent researcher in developing scalable mind-body, mHealth interventions addressing pain and concomitant unhealthy substance use.
- The candidate provided a very detailed and proposed a staged career development plan for each of his stated deficiencies, drawing on scheduled mentoring meetings/supervision with his mentors and advisory board, and formal courses.

Weaknesses

- Some of his core areas of deficiencies overlap and would not seem to require the 5-year timeline as proposed to accomplish.

3. Research Plan:

Strengths

- The significance of the proposed project is that patients with non-traumatic painful upper extremity conditions (NPUC) are highly prevalent and that most patients do not seek medical care. Patients with NPUC, who have a history of risky substance use, tend to seek care more frequently, have higher rates of disability and healthcare utilization, and report higher pain intensity. This observation is based in part by his own past research, and that it is not common practice to assess or manage these cases. A theoretical, mechanistic model of the reciprocal nature of pain and substance use was provided to lay the foundation for the proposed intervention.
- A compelling rationale is provided that mind-body interventions can be efficacious in addressing risky substance use and possibly reduce the pain experience and that the delivery of a mind-body intervention via an asynchronous web-based platform will reduce access barriers, cost, and stigmatization.

- The intervention, TIRELESS, is based on the reciprocal model of pain and substance use and the fear avoidance model and adapted from a vetted intervention, the TOR, developed by his mentors (Drs. Vranceanu and Ring) which he has experience with. The candidate proposes adapting TOR to be delivered asynchronously and not by a trained therapist and focusing on pain and risky substance use. This intervention draws on the candidate's previous research experience and that of his mentors, providing a strong practical and theoretical base for success.
- The research approach is well delineated and includes development and initial pilot testing followed by an intervention phase of a single-blind feasibility RCT comparing TIRELESS to a web-based usual care control acquiring data on acceptability and feasibility as the basis to apply for a R01 award for full testing.
- The study design is described in detail and is comprehensive and demonstrates good scientific rigor.
- Potential challenges to the approach and alternative strategies are thoughtful and well delineated. For example, one strategy is budgeting for smart phones if subjects have no or limited access to technology.
- In general, the proposed assessments are reasonable, with the exception of the assessment of opioid misuse (see weaknesses below).
- The power analysis and statistical plan, patient engagement, and protocol adherence are all appropriate and well described.

Weaknesses

- Using the COMM to detect aberrant drug-related behavior and changes in COMM as a marker of improvement can be problematic. The COMM is well validated for use in patients receiving long-term opioid therapy but no other potential drugs of abuse/misuse (alcohol, cocaine, illicit opioids, nicotine). The TLFB is an assessment tool for all drugs, alcohol, and nicotine but is based on patient recall and subjectivity.
- The lack of more objective assessments of misuse (e.g., EMR, urine drug testing, and PDMP).
- The target population is poorly described with respect to prevalence, in other words, it is not clear if there is a large enough population with non-traumatic UE pain and co-morbid substance misuse to warrant further study.

4. Mentor(s), Co-Mentor(s), Consultant(s), Collaborator(s):
Strengths

- The mentoring team is exceptional. His primary mentor, Dr. Vranceanu, has an impressive record in obtaining grant support and throughout her career has been devoted to mentorship, typically overseeing two PhD degree-level psychologists, four post-doctoral fellows, and has developed the adapted intervention for the candidate's proposed research project. Drs. Vranceanu and Bakhshaie have published six peer-reviewed publications together, mostly in the proposed area of the candidate's research interests.
- Dr. Ditre is a nationally known expert in the intersection of pain and SUD, in particular his seminal work is on pain and smoking. He has also been a mentor of Dr. Bakhshaie; they have published peer-reviewed publications together, and he will provide support in clinical trial and data management, grant writing, and career development.
- Dr. Levy is a health services researcher who would provide guidance on the process of health care utilization and cost data analysis; Dr. Chen is a hand and UE orthopedic surgeon who would provide support with recruitment; and Dr. Ring is an orthopedic surgeon at the University of Texas at Austin with expertise in the relationship of mental health and disability. Finally, Dr. Sylvia provides expertise in web-based pragmatic trials and would help the candidate to develop the TIRELESS intervention.
- Overall, this is a strong mentoring team, many of whom have worked directly with the candidate and provide expertise to ensure the success of his candidate and his proposed project.

Weaknesses

- The mentoring team may have been strengthened with the addition of an expert for direct mentoring on mixed methods design and analysis to supplement his previous training in biostatistics rather than relying on access to the Biostat Center and other course work.

5. Environment:
Strengths
- MGH has exceptional resources to support the development of the candidate into an independent researcher, including a strong telehealth department, a Center for Integrative Medicine (aligns with his stated research goals of developing behavioral health services via telehealth), a strong biostatistical department to support his training in data analysis, a separate center for faculty and the Harvard Catalyst Center to support career development and develop grant writing skills.
- The candidate's main mentor, Dr. Vranceanu, recruited the candidate and works closely with him and appears in a position to protect his time.
- The application budget provides the candidate 9.0 calendar months to devote to the proposed project with additional funds from other grant awards to allow him the latitude to develop needed skills to be successful as an independent researcher.

Weaknesses
- No major weaknesses noted.

Study Timeline:
Strengths
- The project timeline is very detailed and realistic in meeting the stated objectives of this project.
- Contingency plans to account for unexpected challenges for recruitment, patient engagement, and retention are adequate.
- The candidate has a well-established relationship with his primary mentor and has described a plan that utilizes the resources of MGH/Harvard University.

Weaknesses
- No major weaknesses noted.

Protections for Human Subjects
Acceptable Risks and Adequate Protections
- Protections for human subjects are well described and appropriate.

Data and Safety Monitoring Plan (Applicable for Clinical Trials Only):
Not Applicable (No Clinical Trials)

Inclusion Plans
- Sex/Gender: Distribution justified scientifically
- Race/Ethnicity: Distribution justified scientifically
- For NIH-Defined Phase III trials, Plans for valid design and analysis: Not applicable
- Inclusion/Exclusion Based on Age: Distribution justified scientifically
- Given the study population on degenerative orthopedic disorders, younger patients (<18 years old) will be excluded and there is no upper age limit exclusions.

Vertebrate Animals
Not Applicable (No Vertebrate Animals)

Biohazards
Not Applicable (No Biohazards)

Training in the Responsible Conduct of Research
Acceptable

Comments on Format:
- The format is acceptable.

Comments on Subject Matter:
- The subject matter is acceptable.

Comments on Faculty Participation:
- Faculty participation is acceptable.

Comments on Duration:
- The duration is acceptable.

Comments on Frequency:
- The frequency is acceptable.

Select Agents
Not Applicable (No Select Agents)

Resource Sharing Plans
Acceptable

Authentication of Key Biological and/or Chemical Resources
Not Applicable (No Relevant Resources)

Budget and Period of Support
Recommend as Requested

CRITIQUE 2

Candidate: 2
Career Development Plan/Career Goals /Plan to Provide Mentoring: 3
Research Plan: 5
Mentor(s), Co-Mentor(s), Consultant(s), Collaborator(s): 2
Environment Commitment to the Candidate: 1

Overall Impact: This is a K23 application to provide the candidate with additional training to prepare them for a career in academic research. The application includes a research plan, which consists of three aims; qualitative interviews; an open, non-controlled pilot; and a formal feasibility randomized controlled clinical trial (RCT). The candidate is extremely well published and very productive. Their career development plan is well organized and comprehensive. The only concerns noted are whether this much additional training is needed given the extensive experiences already acquired. The mentors and the training environment are outstanding. The research plan seems methodologically sound, but the overall significance and premise of targeting this seemingly narrow subset of a population is not fully justified. The discussion of other treatment options for this group lacks detail and the jumping straight to telehealth interventions is not fully explained. Proposing health economic assessment and training at this stage seems premature.

1. Candidate:
Strengths
- The candidate is well-trained in both medicine and in clinical psychology. He also has some expertise in statistics.
- The candidate has a very strong record of publication with over 100 publications, many as first author.
- The candidate has solid experience in substance use treatment and research.
- The candidate has experience studying health inequalities and underserved populations.

- The candidate has considerable training around the role that emotional issues may play in substance use and the expression of other medical conditions.
- The candidate has experience managing large research projects.

Weaknesses

- No major weaknesses noted.

2. Career Development Plan/Career Goals & Objectives:

Strengths

- The career development plan provides clear outlines of training goals and coursework to address his four goals.
- Training will occur in four main areas: qualitative and mixed method research, mind-body clinical trials in orthopedics, mobile (m)Health methods, and economic evaluation methods.
- Long-term career goals are consistent with the proposed training and are clearly described.
- An extensive array of coursework is described to address these goals.
- Current skills and skill gaps are carefully outlined and the training activities to address these gaps are also well described.

Weaknesses

- Given the extensive research experience that the candidate has, and 134 publications, just because they have not independently led aspects of research as described in B. Training Goals, does not mean they need extensive training in these areas. It seems some of the gaps in the first three training goal areas may be overstated.
- Likewise, the current mentors of the candidate are experts in many of these training areas and much of this training could likely develop without a K award.

3. Research Plan:

Strengths

- Non-traumatic upper extremity conditions are likely prevalent and risky substance use appears to be a co-occurring problem.
- Given there is adequate time, the open pilot may provide additional information for refining this program.
- The Toolkit for Optimal Recovery (TOR) after injury, on which the new intervention will be based, is well described.
- The feasibility benchmarks for Aim 3 are well described.

Weaknesses

- The application does not take the time to establish the impact and prevalence of the target population. It is unclear how many adults experience significant enough non-traumatic painful upper extremity conditions (NPUC) to seek care or be referred to specialty clinics and also have risky substance use. It is acknowledged that many or most people with NPUC don't need surgery and don't have risky substance use. It is stated that NPUCs are "common", yet the main reference cited on rotator cuff problems provides no prevalence data.
- Usual medical care for this population is not well described and seems oversimplified. Surgeries, medication, and physical therapy (PT) are all mentioned together, or medical care is described as largely palliative, e.g., injections, pain medications. Stepped care and the use of PT are not explicitly mentioned, and it is unclear at what stage the proposed intervention might best be used (e.g., after PT but before surgery).
- Risky substance use is described as a "maladaptive pain behavior" but this seems an oversimplification. It could pre-date the NPUC and/or be unrelated. It could also have potentially directly led to the NPUC from past minor injury or use, or sometimes it may be a maladaptive response to the NPUC. Thus, it is just unclear how often this is the case.
- The Research Plan section of the application talks about risky substance use but it does not exclude those who have a primary substance use disorder (SUD). Given the challenges and low

response rate to intensive SUD treatment programs, it seems hard to justify using an asynchronous online program with those with serious SUDs.
- There is growing evidence that cannabis may be a useful medical treatment for chronic pain, and it is a legal treatment in many states. This issue and its use as inclusion criteria should be discussed.
- The limitations of the use of other psychological or mind-body programs for this population is not adequately discussed. The Candidate Information section of the application mentioned learning about the "limitations of traditional cognitive behavioral approaches", but these are not made explicit. The idea of avoiding stigma around substance use is briefly mentioned.
- It is unclear why existing mind-body interventions for chronic pain are not used. They can also avoid stigmatization and substance use is also relevant in other chronic pain conditions.
- The justification for jumping to an asynchronous telehealth intervention is lacking. Stigma is mentioned, but there is also concern that social connection and interpersonal aspects of individual or group sessions can also lessen effects for some people. Having the intervention materials for the future can be accomplished through paper or online materials and resources for any intervention. As the pandemic has waned, many people are seeking in-person connections because of the effects on mood from social isolation.
- The methods for accessing the electronic heath record (EHR) and quantifying health care utilization or costs are not clearly described.
- The economic analyses seem quite premature when preliminary qualitative work has yet to be conducted and two pilot studies must first be conducted. Establishing some signal of feasibility and potential effectiveness should occur first.

4. Mentor(s), Co-Mentor(s), Consultant(s), Collaborator(s):
Strengths
- Dr. Ring is a well-known academic researcher with extensive mentoring experience.
- Dr. Vranceanu has emerged as a leading mind-body intervention and pain researcher.
- Other mentors such as Drs. Levy and Sylvia are highly qualified and appropriate.
- Strong recommendations letters are provided.

Weaknesses
- No major weaknesses noted.

5. Environment:
Strengths
- Massachusetts General Hospital (MGH) is an excellent clinical research training environment for the candidate.
- The candidate's role at the Center for Health Outcomes and Interdisciplinary Research (CHOIR) also offers a ready-made environment for training and learning.
- The candidate has access to training through many different programs including the Osher Center, the Benson Henry Institute (BHI), and the MGH Institute for Technology.

Weaknesses
- No major weaknesses noted.

Study Timeline:
Strengths
- The career development program is associated with a timeline.
- A timeline is provided for the research studies.

Weaknesses
- The career development timeline is a little hard to follow but is adequate.

Protections for Human Subjects
Acceptable Risks and Adequate Protections

Data and Safety Monitoring Plan (Applicable for Clinical Trials Only):
- Acceptable

Inclusion Plans
- Sex/Gender: Distribution justified scientifically
- Race/Ethnicity: Distribution justified scientifically
- For NIH-Defined Phase III trials, Plans for valid design and analysis: Not applicable
- Inclusion/Exclusion Based on Age: Distribution justified scientifically
- All areas are addressed and appear justified.

Vertebrate Animals
Not Applicable (No Vertebrate Animals)

Biohazards
Not Applicable (No Biohazards)

Training in the Responsible Conduct of Research
Acceptable
Comments on Format:
- The format seems good and includes online and in-person sessions.

Comments on Subject Matter:
- The breadth and content of subject matter are good.

Comments on Faculty Participation:
- Mentors are involved in training.

Comments on Duration:
- The duration meets the requirements.

Comments on Frequency:
- The plan meets the frequency requirements.

Resource Sharing Plans
Acceptable

Budget and Period of Support
Recommend as Requested

CRITIQUE 3

Candidate: 1
Career Development Plan/Career Goals /Plan to Provide Mentoring: 1
Research Plan: 2
Mentor(s), Co-Mentor(s), Consultant(s), Collaborator(s): 1
Environment Commitment to the Candidate: 1

Overall Impact: The candidate aims to become an independent research scientist with a program of clinical research focused on developing and testing scalable, technology-enhanced, and cost-effective mind-body interventions addressing pain and substance use and orthopedic and other costly musculoskeletal conditions across multiple settings. The candidate's career development goals include developing skills in qualitative and mixed methods, the conduct of mind-body clinical trials with orthopedic patients, the development of asynchronous web-based platforms and mobile (m)Health approaches, and economic evaluation. The application aims to adapt an existing intervention into a brief, integrated mind-body program, developed for delivery via an asynchronous web-based platform,

to provide support to patients with comorbid risky substance use and nontraumatic painful upper extremity conditions. Enthusiasm is high for this application due to a highly promising candidate with a substantial history of productivity; a novel area of focus on nontraumatic upper extremity pain conditions; an outstanding mentorship team and environment and a well-conceived approach. Taken together, the likelihood of the application leading to the candidate's goal of becoming a successful independent investigator is high.

1. Candidate:
Strengths
- The candidate has an extraordinary number of publications for his stage of career.
- Outstanding reference letters highlight the candidate's potential to become an independent investigator, his professional maturity, motivation, and collegiality.
- The candidate has substantial clinical experience in mind-body interventions to support this research as a clinical psychologist.
- His work as research staff has prepared him well for this application.
- The candidate has a strong background in advanced statistics from his doctorate.
- The candidate has developed a novel instrument to measure anxiety sensitivity.
- The candidate has experience in health disparities in the Latin X community in particular.
- He has been recognized by his institution and promoted from staff psychologist to assistant professor.
- His track record in publications, prior research experience, and prior training supports, as well as his commitment to becoming an independent investigator and his potential to implement the proposed studies are viewed as a strength.

Weaknesses
- A minor weakness in one area of his career goals and objectives reflects potential bias, stating that: "Economic evaluation methodology and analysis so that I can ultimately show that mind-body approaches…are effective…".

2. Career Development Plan/Career Goals & Objectives:
Strengths
- The candidate has identified explicit new areas of knowledge that are required to meet his goals, specifically mind-body practices for pain, the design and conduct of clinical trials, and qualitative methods in order to refine interventions.
- The set of society meetings and intensive trainings to complement his mentoring are appropriate.
- The proposed mentoring meeting frequency is appropriate.
- The proposed training goals and skill gaps are well articulated.
- Adequate plans for monitoring his progress through regular, twice annual meetings of his entire mentorship team are in place.

Weaknesses
- No major weaknesses noted.

3. Research Plan:
Strengths
- The application contains a logical sequence of aims that correspond to the National Center for Complementary and Integrative Health (NCCIH)'s research development sequence.
- There is an appropriate conceptual model for the project.
- The choice of feasibility outcomes for the pilot randomized controlled clinical trial (RCT) is appropriate.
- The choice of measures for efficacy are appropriate.
- Upper extremity pain syndromes are understudied from the perspective of nonpharmacologic therapies and is therefore innovative.

- The adaptation of an existing intervention from the candidate's mentor (the Toolkit for Optimal Recovery) to the context of upper extremity pain syndromes with risky behaviors for substance use is viewed as a strength.

Weaknesses
- The candidate states that 20% of the patients from the orthopedic clinic have risky substance use. However, it is unclear how that estimate was made.
- It is unclear who is creating the sequence of randomization. In one place of the application, it appears that the candidate will be doing the sequence of randomization. In another place in the application, it appears that the biostatistician will be the one responsible for randomization, which is more appropriate.
- The plans for dealing with potential problems are not well detailed.

4. Mentor(s), Co-Mentor(s), Consultant(s), Collaborator(s):
Strengths
- The application includes an outstanding team of mentors.
- The primary mentor has received many mentoring awards and currently has a K24 award providing protected time for mentorship. The mentor has started a national virtual K club which the candidate will benefit from. The mentor and her team have high academic productivity with 15-25 publications per year.
- The collaborator, Dr. Ditre has experience at the intersection of pain and substance use which is the focus of the candidate's project.
- Appropriate mentorship is also in place with health economics analysis expertise, and orthopedic surgeons who focus on upper extremity syndromes with strong research backgrounds.
- Orthopedic researchers with a strong background and interest in biopsychosocial approaches is notable.
- Dr. Sylvia brings expertise in brief online asynchronous interventions.

Weaknesses
- No major weaknesses noted.

5. Environment:
Strengths
- Outstanding research facilities, resources, and training opportunities are available.
- There is strong institutional commitment to the candidate.

Weaknesses
- No major weaknesses noted.

Study Timeline:
Strengths
- A well-developed, feasible timeline is included.

Weaknesses
- Potential challenges and corresponding solutions are not well delineated.

Protections for Human Subjects
Acceptable Risks and Adequate Protections
Data and Safety Monitoring Plan (Applicable for Clinical Trials Only):
- Acceptable

Inclusion Plans
- Sex/Gender: Distribution justified scientifically
- Race/Ethnicity: Distribution justified scientifically
- For NIH-Defined Phase III trials, Plans for valid design and analysis: Not applicable

- Inclusion/Exclusion Based on Age: Distribution justified scientifically

Vertebrate Animals
Not Applicable (No Vertebrate Animals)

Biohazards
Not Applicable (No Biohazards)

Training in the Responsible Conduct of Research
Acceptable
Comments on Format:
- The format is acceptable.

Comments on Subject Matter:
- The subject matter is acceptable.

Comments on Faculty Participation:
- Faculty participation is acceptable.

Comments on Duration:
- The duration is acceptable.

Comments on Frequency:
- The frequency is acceptable.

Resource Sharing Plans
Acceptable

Budget and Period of Support
Recommend as Requested

CRITIQUE 4

Overall Impact: This K23 application proposes to identify treatment needs and preferences of adult patients with non-traumatic painful upper extremity conditions and comorbid risky substance use; develop an asynchronous web-based intervention (TIRELESS) to address pain and substance use in this population; conduct an open pilot of the intervention; and then conduct a feasibility randomized controlled clinical trial (RCT) comparing web-TIRELESS to minimally enhanced usual care. Dr. Bakhshaie is an outstanding candidate, with a record of over 100 peer-reviewed publications and exceptional reference letters. Although he already evidences strong research productivity, he clearly outlined several areas where he would benefit from further training including namely, in qualitative research, mind-body clinical trials in orthopedics, web-based and mobile (m)Health approaches, and economic evaluation. This project addresses highly significant public health issues of risky substance use comorbid with pain in orthopedic populations, and the proposed asynchronous web-based intervention modality has strong potential for scalability. A rigorous pilot trial is proposed, which includes a control condition, a priori specified benchmarks for feasibility, and six-month follow-up. The mentorship team and environment are outstanding. Primary weaknesses pertain to the research plan. First, more detail is needed on how the intervention would be developed and refined (e.g., how might the intervention draw from content in other existing interventions; how are the mentors and other team members involved in the intervention development). Relatedly, it is unclear how the intervention would be tailored to people with upper extremity orthopedic conditions. The application mentions that it could later be adapted to address substance use in the context of other painful orthopedic conditions (e.g., knee, hip, ankle, spine), however, more rationale is needed on why an intervention specific to the population of people with upper extremity conditions is required (e.g., how would it differ from similar interventions for other orthopedic populations). Second, the sample would be quite heterogeneous with

regard to substance use (i.e., alcohol, cannabis, cocaine, opioids, tobacco products, etc.). The intervention may need to be different depending on substance type, and this does not seem to be considered. If the intervention does target such a wide variety of substances, it would be important to include sufficient heterogeneity in the pilot work, and that may not be feasible given the small sample sizes. Third, the assessment plan does not include any biochemical confirmation of substance use. Fourth, although data on medical care utilization and expenditures will be obtained in the pilot trial, this part of the application is underdeveloped. This seems to be a missed opportunity given the focus on economic evaluation methodology as one of the training goals. Finally, the career development plan is ambitious, but this is a minor concern given the incredible productivity of this promising scholar. Overall, this is a highly significant application with strong promise to further the candidate's already impressive research career.

THE FOLLOWING SECTIONS WERE PREPARED BY THE SCIENTIFIC REVIEW OFFICER TO SUMMARIZE THE OUTCOME OF DISCUSSIONS OF THE REVIEW COMMITTEE, OR REVIEWERS' WRITTEN CRITIQUES, ON THE FOLLOWING ISSUES:

PROTECTION OF HUMAN SUBJECTS: ACCEPTABLE

INCLUSION OF WOMEN PLAN: ACCEPTABLE

INCLUSION OF MINORITIES PLAN: ACCEPTABLE

INCLUSION ACROSS THE LIFESPAN: ACCEPTABLE

TRAINING IN THE RESPONSIBLE CONDUCT OF RESEARCH: ACCEPTABLE

COMMITTEE BUDGET RECOMMENDATIONS: The budget was recommended as requested.

Footnotes for 1 K23 AT012364-01; PI Name: Bakhshaie, Jafar

NIH has modified its policy regarding the receipt of resubmissions (amended applications).See Guide Notice NOT-OD-18-197 at https://grants.nih.gov/grants/guide/notice-files/NOT-OD-18-197.html. The impact/priority score is calculated after discussion of an application by averaging the overall scores (1-9) given by all voting reviewers on the committee and multiplying by 10. The criterion scores are submitted prior to the meeting by the individual reviewers assigned to an application, and are not discussed specifically at the review meeting or calculated into the overall impact score. Some applications also receive a percentile ranking. For details on the review process, see http://grants.nih.gov/grants/peer_review_process.htm#scoring.
